# Eating Habits, Body Weight Perception, and Psycho-Emotional Factors Among Romanian University Students: A Cross-Sectional Study

**DOI:** 10.3390/nu18121837

**Published:** 2026-06-06

**Authors:** Ramona Amina Popovici, Baleanu Vlad-Dumitru, Laria-Maria Trusculescu, Andreea Mihaela Kiș, Alexandra Enache, Cristina Raluca Bodo, Ana Gabriela Seni, Liana Dehelean, Anca Porumb, Diana Marian, Alexandru Mischie, Dana Emanuela Cot (Pitic), Adina Feher, Liana Todor

**Affiliations:** 1 Faculty of Dental Medicine, “Victor Babes” University of Medicine and Pharmacy, 300041 Timisoara, Romania; ramona.popovici@umft.ro (R.A.P.); laria.trusculescu@umft.ro (L.-M.T.); dana.pitic@umft.ro (D.E.C.); 2 Department of General Surgery, Faculty of Medicine, ‘Carol Davila’ University of Medicine and Pharmacy, 020021 Bucharest, Romania; vlad.baleanu@umfcd.ro; 3 Surgery Department, Clinical Emergency Hospital Sf. Pantelimon, 340–342 Pantelimon Road, 021659 Bucharest, Romania; 4 Department of Neuroscience, Discipline of Forensic Medicine, Bioethics, Deontology and Medical Law, “Victor Babes” University of Medicine and Pharmacy, 300041 Timisoara, Romania; 5 Department of Bioethics, Medical Deontology and Medical Communication, “George Emil Palade” University of Medicine, Pharmacy, Science and Technology of Târgu Mureș, 540139 Târgu Mureș, Romania; cristina.bodo@umfst.ro; 6 Doctoral School, “George Emil Palade” University of Medicine, Pharmacy, Science and Technology of Târgu Mureș, 540139 Târgu Mureș, Romania; gabriela.seni@umfst.ro; 7 Neuroscience Department, Faculty of Medicine, “Victor Babes” University of Medicine and Pharmacy, 300041 Timisoara, Romania; lianadeh@umft.ro; 8 Dental Medicine Department, University of Oradea, 410068 Oradea, Romanialiana.todor@uoradea.ro (L.T.); 9 Department of Dentistry, Faculty of Dentistry, “Vasile Goldiș” Western University of Arad, 94–96 Revolutiei Blvd., 310025 Arad, Romania; marian.diana@uvvg.ro; 10 Doctoral School, “Victor Babes” University of Medicine and Pharmacy, 300041 Timisoara, Romania; alexandru.mischie@umft.ro (A.M.); adina.feher@umft.ro (A.F.)

**Keywords:** body image distortion, eating disorders, nutritional literacy, academic stress, Romania

## Abstract

Introduction: Dietary habits adopted during young adulthood play a critical role in physical, emotional, and cognitive health. University students represent a particularly vulnerable group due to academic stress, lifestyle transitions, and increased autonomy, factors that may influence eating behaviors, body weight perception, and psychological well-being. This study aims to examine dietary habits among students and their associations with self-perceived body weight, lifestyle characteristics, and psychological factors within a biopsychosocial framework. Materials and Methods: A cross-sectional observational study was conducted using a structured, self-administered online questionnaire distributed to university students aged 18–30 years in Romania. The questionnaire assessed dietary habits, nutritional knowledge, lifestyle behaviors, and psychological variables, including perceived stress and body weight perception. Body mass index was calculated based on self-reported anthropometric data. Results: The findings indicated substantial variability in dietary behaviors, with a high prevalence of irregular meal patterns, frequent snacking, and engagement in weight-control practices. Irregular meal patterns were reported by approximately 62% of participants, while 47% had engaged in at least one weight-loss diet. Discrepancies between self-reported BMI and perceived body weight were observed in roughly 38% of cases, and 83% of respondents reported at least one psychological symptom (stress, anxiety, or low mood) related to eating behaviors. A positive correlation was observed between sleep duration and perceived rest quality (r = 0.364, *p* < 0.001). High frequencies of caffeinated beverage consumption were also observed. Additionally, 204 participants reported no alcohol consumption, while the variety of alcoholic beverages consumed was strongly correlated with alcohol intake frequency (r = 0.734, *p* < 0.001). Conclusions: Dietary habits among university students are closely interconnected with body weight perception, lifestyle behaviors, and psychological well-being. These findings emphasize the need for integrative health promotion strategies that address nutrition, emotional regulation, and lifestyle balance to support mental and cognitive health during young adulthood.

## 1. Introduction

Dietary habits established during young adulthood play an important role in long-term physical, emotional, and cognitive health. University students experience significant lifestyle changes, including increased independence, academic stress, social pressure, and irregular daily routines, all of which may influence eating behaviors. Recent research suggests that nutrition affects not only metabolic health, but also emotional well-being, cognitive performance, and mental health through complex neurobiological and psychosocial mechanisms.

Unhealthy dietary patterns among students—including meal skipping, frequent consumption of ultra-processed foods, high intake of sugar and saturated fats, and excessive use of stimulants or alcohol—have been associated with poorer academic performance, higher stress levels, anxiety, depressive symptoms, and impaired sleep quality [1,2,3,4,5,6]. These behaviors are common among students, where academic demands and time constraints often lead to irregular eating schedules and convenience-based food choices. In contrast, students who maintain regular meals and healthier dietary habits tend to show better concentration, memory, and academic performance [2].

From a biological perspective, dietary factors exert a significant influence on brain function via mechanisms involving neuroinflammation, oxidative stress, insulin resistance, and dysregulation of neurotransmitter systems. Metabolic dysregulation associated with obesity and diabetes (“diabesity”) further contributes to systemic inflammatory burden and neurocognitive vulnerability [7,8]. Distorted weight perception, either overestimation or underestimation of one’s own body weight, has been linked to disordered eating behaviors, low self-esteem, and maladaptive coping strategies, including restrictive dieting or compulsive eating [8,9].

Nutritional knowledge and self-management skills have emerged as important moderators of eating behavior and body weight perception among university students. Higher levels of nutrition-related knowledge are associated with healthier food choices, lower dietary fat intake, and improved awareness of portion control [9,10]. Traditional Romanian dietary patterns are characterized by high consumption of meat products, bread, and calorie-dense foods, while adherence to healthy nutrition recommendations remains inconsistent, especially among young adults [11,12]. Recent studies conducted among Romanian university students have highlighted concerns regarding unhealthy dietary patterns, limited food literacy, and weight-related problems, emphasizing the growing importance of nutrition research within higher education institutions [13,14,15]. Although nutrition science and public health education have expanded in Romanian universities in recent years, evidence regarding students’ dietary habits and associated lifestyle factors remains limited, supporting the need for further country-specific investigations [14,15]. Similarly, studies conducted among Romanian dental students have revealed variability in knowledge, attitudes, and preventive practices regarding infectious disease risk, highlighting the importance of educational strategies to promote responsible health behaviors [16].

The university environment may further amplify these discrepancies through social comparison, media exposure, and cultural norms that emphasize thinness or muscularity as standards of success and attractiveness. At the same time, extreme or unbalanced dietary practices, often adopted in response to negative body weight perception, may exacerbate nutritional deficiencies, disrupt sleep, and impair emotional regulation [17,18,19].

Physical activity is another key determinant of the interaction among diet, emotional well-being, and cognitive health during young adulthood. Sedentary behavior, highly prevalent among university students, is associated with increased cardiovascular risk, metabolic dysfunction, and impaired cognitive performance [20,21,22]. Regular physical activity has been shown to exert neuroprotective effects by improving cerebral blood flow, reducing systemic inflammation, and enhancing executive function, memory, and emotional regulation [23,24]. Importantly, dietary quality and physical activity act synergistically, as inadequate nutrition may limit exercise capacity, while physical inactivity may exacerbate weight gain and negative body image. Dietary behaviours during youth may also influence broader developmental outcomes, including somatic growth, cognitive performance, and oral health indicators [25]. In student populations, barriers such as academic workload and perceived lack of time further contribute to reduced engagement in physical activity, amplifying both psychological distress and unhealthy eating behaviors [26].

Despite extensive international research on diet, mental health, and cognition, studies simultaneously examining lifestyle factors, psychological well-being, nutritional knowledge, and self-perceived body weight in student populations remain limited, particularly in Eastern European contexts. Moreover, few investigations adopt an integrative framework that captures both the behavioral and perceptual dimensions of nutrition-related health in young adulthood.

The increasing popularity of restrictive and trend-driven dietary strategies among young adults further complicates the relationship between nutrition, emotional health, and cognitive functioning. Diets such as intermittent fasting, ketogenic regimens, paleo diets, and detoxification protocols are frequently adopted by students in pursuit of rapid weight loss or improved self-image, often without medical supervision [27]. While some approaches may confer short-term metabolic benefits, evidence regarding their long-term cognitive and psychological effects remains mixed and, in some cases, controversial [28,29,30,31]. Extreme dietary restriction has been associated with heightened stress responses, impaired concentration, sleep disturbances, and an increased risk of binge-eating behaviors following dietary cessation [32,33]. Given the emotional vulnerability of the student population, the widespread promotion of such diets through social media and peer networks raises concerns regarding their potential neurocognitive and psychological consequences.

By situating eating behavior within a broader biopsychosocial context, this research seeks to contribute to a more comprehensive understanding of how dietary patterns intersect with emotional and cognitive health during a formative life stage [34].

The present study aimed to investigate the associations between dietary behaviours, body weight perception, psychological well-being, and selected lifestyle factors among university students. We hypothesized that irregular eating patterns and restrictive dietary practices are associated with increased psychological distress, negative body image perception, sleep disturbances, and maladaptive health behaviours. By exploring these multidimensional relationships within a biopsychosocial framework, this study aims to better understand how nutrition-related behaviours interact with emotional and cognitive health in young adult populations.

## 2. Materials and Methods

### 2.1. Study Design and Participants

This study employed a cross-sectional observational design to analyze dietary habits among university students and their associations with self-perceived body weight, lifestyle characteristics, and psychological factors. Data were collected using a structured, self-administered questionnaire distributed online. Data collection was conducted between December 2025 and February 2026. The target population consisted of young adults enrolled in higher education institutions in Romania.

A total of 380 participants were included in the study. Eligibility criteria comprised age between 18 and 30 years and current or recent enrollment in a university-level educational program. No restrictions were imposed regarding field of study, sex, or area of residence (urban or rural). Participants who did not fall within the specified age range were excluded from the analysis. Participation was voluntary, and no financial or material incentives were offered.

### 2.2. Data Collection Instrument

Data were collected using a custom-designed questionnaire developed specifically for this study. The instrument consisted of 39 items grouped into four main sections: The complete questionnaire is provided as Supplementary Materials

1. General and anthropometric data;
2. Lifestyle characteristics;
3. Dietary habits and nutritional knowledge;
4. Psychological and perceptual factors.

The questionnaire was designed to be concise, easy to understand, and suitable for online completion. General data included age, sex, area of residence, field of study, self-reported height and weight, and personal medical history. Body mass index (BMI) was calculated based on self-reported anthropometric data using the standard formula (kg/m^2^). Lifestyle-related questions assessed physical activity level, sleep duration and perceived sleep quality, caffeine and alcohol consumption patterns, and smoking status. Dietary-related items addressed meal frequency, snacking behavior, type of diet followed, attention to food labels, calorie monitoring practices, and prior engagement in weight-loss diets. Nutritional knowledge was assessed using ordinal response categories (advanced/moderate/no knowledge) to indicate self-perceived understanding of macronutrients and micronutrients, rather than through objective knowledge testing. Psychological and perceptual dimensions included satisfaction with body image, perceived body weight, self-esteem indicators, perceived stress, anxiety, depressive symptoms, and perceived social and familial support.

All responses were self-reported and recorded using multiple-choice selections, Likert-type scales, or short numerical inputs, depending on the nature of the variable assessed.

The questionnaire was distributed electronically via online platforms and social media channels commonly used by students. Before participation, all respondents were informed about the purpose of the study, the voluntary nature of participation, and the anonymous processing of data. Informed consent was obtained electronically before the questionnaire could be accessed. The estimated completion time for the questionnaire was approximately 10–15 min.

The questionnaire was specifically developed for this study based on previously published instruments investigating dietary habits, body image perception, lifestyle behaviours, and psychological well-being among young adults [1,2,3,4,5,6,8,9,10,35]. Because the instrument combined adapted and newly formulated exploratory items, it should not be considered a formally validated psychometric tool. A pilot test involving 25 university students was conducted to assess clarity, comprehensibility, and completion time, leading to minor wording adjustments.

No major structural changes to the instrument were required following the pilot phase, and the final version was subsequently used for data collection. However, the psychological variables were assessed through self-reported perceptions rather than standardized clinical diagnostic tools. The pilot phase was conducted exclusively to evaluate clarity, comprehensibility, item wording, and estimated completion time, and not to perform formal psychometric validation of the questionnaire.

To minimize missing data, all key variables were required. However, participants retained the right to withdraw from the study at any point by discontinuing questionnaire completion.

### 2.3. Statistical Analysis

Data analysis was conducted using IBM SPSS Statistics software, version 26.0 (IBM Corp., Armonk, NY, USA). Descriptive statistics were used to summarize demographic characteristics, dietary behaviors, lifestyle factors, and psychological variables. Continuous variables were expressed as means and standard deviations, while categorical variables were presented as frequencies and percentages.

An a priori power analysis was conducted to estimate the minimum required sample size for the correlational analyses performed in this study. Assuming a significance level of α = 0.05, a statistical power of 1 − β = 0.80, and an expected small-to-moderate effect size (r = 0.20–0.30), the estimated minimum sample size ranged between approximately 190 and 320 participants. Therefore, the final sample of 380 participants was deemed sufficient to provide adequate statistical power to detect significant associations among dietary, lifestyle, and psychological variables.

Associations between dietary habits, body weight perception, lifestyle variables, and psychological factors were examined using Spearman’s rank correlation coefficient, chosen because several variables were ordinal and no assumptions about normality were required. Statistical significance was set at a *p*-value < 0.05. Due to the exploratory nature of the study and the ordinal nature of several variables, only Spearman correlations were performed; multivariate analyses (e.g., logistic or linear regression) were not conducted to avoid overfitting in this cross-sectional design. Effect sizes were interpreted following Cohen’s conventions for correlation coefficients: small (|r| < 0.30), medium (0.30 ≤ |r| < 0.50), and large (|r| ≥ 0.50). Given the number of correlations examined and the absence of formal correction for multiple comparisons (e.g., Bonferroni or false discovery rate), the risk of Type I error should be considered when interpreting borderline findings; results should therefore be regarded as hypothesis-generating rather than confirmatory.

Due to ethical considerations related to participant confidentiality and the use of self-reported health-related data, the dataset has not been deposited in a publicly accessible repository. No restrictions apply to the analytical methods or questionnaire structure, which can be shared for replication purposes.

### 2.4. Ethical Considerations

The study was conducted in accordance with the principles of the Declaration of Helsinki. Participation was voluntary, anonymous, and based on informed consent obtained electronically before completing the questionnaire. The study protocol, including the data collection instrument and procedures, was reviewed and approved by the University’s Ethics Committee.

## 3. Results

### 3.1. General and Anthropometric Data

A total of 380 university students aged 18–30 years were included in the analysis. Of these, 226 (59%) were female, and 154 (41%) were male. Most participants originated from urban areas (n = 302, 79%), while 78 (21%) were from rural areas (Table 1). Percentages presented in the tables were calculated relative to the total study population (n = 380).

Participants were categorized into five age groups: <20 years, 20–21 years, 22–23 years, 24–25 years, and ≥26 years. The largest proportion of respondents was under 20 years of age, whereas the 22–23-year age group had the lowest representation (Table 2).

### 3.2. Lifestyle Characteristics

#### 3.2.1. Physical Activity

Light physical activity (1–2 times/week) was reported by 172 participants, while 102 engaged in moderate activity (3–4 times/week). Seventy-eight participants were classified as sedentary, and 29 reported daily intense physical activity.

A positive association was identified between the number of daily meals and physical activity frequency, indicating that higher physical activity levels were associated with more meals.

Regarding exercise preferences, 57% preferred light activities (e.g., walking), 25% moderate exercise (e.g., jogging), and 17% intense exercise (e.g., bodybuilding, swimming). Intense exercise was more common among male participants.

#### 3.2.2. Caffeine and Alcohol Consumption

Caffeine consumption was highly prevalent, with 285 participants reporting consumption of caffeinated beverages. Natural coffee was the most consumed (n = 227), followed by instant coffee (n = 80), tea (n = 57), and energy drinks (n = 43). Ninety-five participants reported no caffeine intake (Table 3).

Regarding frequency, 148 participants consumed caffeinated beverages once daily, 92 twice daily, and 45 three or more times per day. A strong positive correlation was identified between the diversity of caffeinated beverages consumed and frequency of consumption (r = 0.704, *p* < 0.001).

Several statistically significant correlations were identified among university students between lifestyle behaviours, dietary practices, and maladaptive weight-control behaviours. Strong positive associations were observed between the diversity of alcoholic beverages consumed and alcohol consumption frequency, as well as between dieting practices and self-reported dieting success. Moderate correlations were also identified between sleep duration and perceived rest quality, while weaker but significant associations were observed among restrictive eating behaviours, supplement use, and self-induced vomiting. The main correlations identified in the study are summarized in Table 4.

### 3.3. Psychological and Perceptual Factors

#### 3.3.1. Psychological and Social Influences

A total of 273 participants reported receiving negative comments about their physical appearance. Among them, 170 reported such comments from peers or extended social circles, and 103 from close family members.

A strong positive correlation was identified between receiving negative comments from family and from the broader social environment (r = 0.523, *p* < 0.001) (Table 5).

Most participants (n = 306) reported receiving family support, while 74 did not. An inverse association was identified between family support during dieting and external negative comments (Table 5).

When asked whether they feared weight gain, 181 responded affirmatively and 199 responded negatively.

Most participants reported good adaptability to new situations (n = 289) and the ability to work effectively under pressure (n = 253). The majority (n = 287) reported never having attended psychotherapy.

#### 3.3.2. Body Weight Perception/Body Image Findings

**Figure 1:**
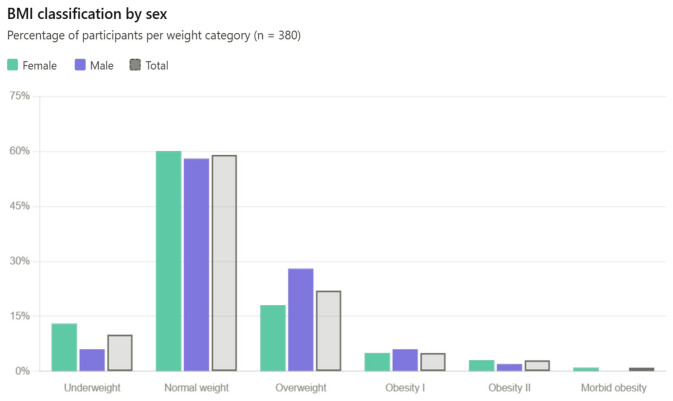
BMI classification according to sex among university students included in the study. Male participants showed a higher prevalence of overweight status, whereas underweight status was more frequent among females.

Based on self-reported height and weight, body mass index (BMI) was calculated for all participants. The mean BMI of the sample was 23.48 kg/m^2^, corresponding to a normal weight classification. Overall, 38 participants (10%) were underweight (BMI < 18.5 kg/m^2^), 226 (59%) were within the normal BMI range, and 116 (31%) exceeded the normal BMI range. Among those above normal weight, 83 (22%) were overweight, 21 (5%) had class I obesity, 10 (3%) had class II obesity, and 2 (1%) had morbid obesity. Detailed anthropometric distributions are presented in Figure 1. BMI values ranged from 14.5 to 50.7 kg/m^2^. Body weight ranged from 40 kg to 150 kg (mean = 69.66 kg), while height ranged from 147 cm to 205 cm (mean = 171 cm).

Regarding medical history, 90% of participants reported no chronic diseases. Among the remaining 10%, the most frequently reported conditions included metabolic disorders (n = 14), cardiovascular diseases (n = 11), endocrine disorders (n = 8), asthma (n = 3), and severe burnout (n = 2).

**Figure 2:**
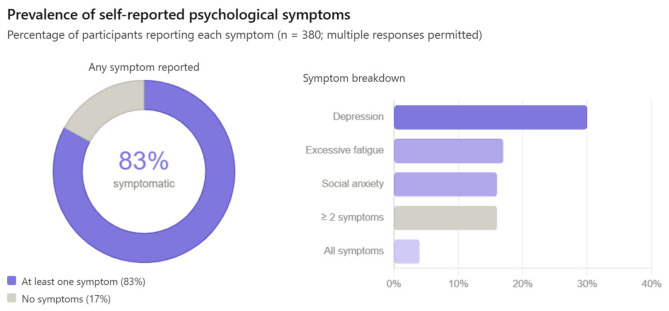
Distribution of self-reported psychological symptoms among study participants.

Psychological symptoms were frequently reported. Depression was indicated by 30% of participants, excessive fatigue by 17%, and social anxiety by 16%. Overall, 83% of respondents reported at least one psychological symptom, and multiple responses were permitted. Sixteen participants reported all listed symptoms, while 59 reported at least two. Sixty-six participants reported none (Figure 2).

Taken together, the results outline a coherent pattern in which dietary irregularity, weight-control practices, lifestyle behaviors (sleep, caffeine, alcohol, physical activity), and psychological indicators (perceived stress, body image, emotional eating) are interrelated rather than operating in isolation. Associations were generally of small-to-moderate magnitude according to Cohen’s conventions, with the stronger correlations clustered within conceptually related domains (e.g., sleep duration and rest quality; alcohol variety and consumption frequency; dieting practice and reported success). Discrepancies between calculated BMI and subjective body weight perception, together with the high prevalence of self-reported psychological symptoms, support a biopsychosocial interpretation of dietary behavior in this Romanian student sample and motivate the integrative discussion that follows.

## 4. Discussion

The present study provides an integrative perspective on dietary habits among university students and their association with body weight perception, lifestyle behaviors, and psychological factors. The findings support the working hypothesis that eating behaviors in young adults cannot be adequately understood in isolation but rather emerge from a complex interaction between nutritional knowledge, emotional state, cognitive demands, and self-perception. This multidimensional framework aligns with growing evidence positioning diet as a key modulator of emotional and cognitive health during early adulthood.

One of the central findings of this study is the high prevalence of irregular eating patterns, including meal skipping and frequent snacking, which has been consistently reported in student populations across diverse cultural contexts. Previous research has demonstrated that such behaviors are associated with reduced academic performance, impaired concentration, and diminished cognitive efficiency [2,35,36]. The findings of the present study are generally consistent with previous research conducted among Romanian university students and other Eastern European populations, which have reported suboptimal dietary habits, irregular meal patterns, and elevated levels of psychological distress among young adults. For example, Savu et al. reported that Romanian students frequently exhibited unhealthy eating behaviors associated with reduced physical activity and poorer overall health status, highlighting the complex interaction between lifestyle factors and well-being in this population [14]. Similar patterns observed in the current study, including associations between dietary irregularities, sleep quality, and psychological symptoms, support the growing evidence that university students in Eastern Europe may represent a vulnerable group in terms of nutrition-related and psycho-emotional health behaviors.

Psychological stress emerged as a prominent factor associated with unhealthy dietary behaviors and negative body weight perception. This observation is consistent with earlier studies indicating that elevated stress levels among university students are linked to increased consumption of energy-dense, palatable foods and disrupted meal timing [3,4]. Chronic stress activates the hypothalamic–pituitary–adrenal axis, leading to increased cortisol secretion, which has been shown to influence appetite regulation and promote visceral fat accumulation.

The relationship between dietary habits and mental health indicators observed in this study further supports previous evidence linking depression and anxiety symptoms to altered food consumption patterns in young adults [5,6]. Emotional distress has been associated with both restrictive and compulsive eating behaviors, highlighting the bidirectional nature of the diet–mental health relationship. Food addiction-like behaviors, particularly toward ultra-processed foods rich in sugar and fat, have been shown to involve reward pathways similar to those implicated in substance use disorders and negatively influence body image [1,8,9].

A particularly relevant contribution of this study lies in the identification of discrepancies between self-reported BMI (calculated from self-reported height and weight) and subjective body weight perception. Consistent with prior research, distorted self-perception was associated with unhealthy eating behaviors, increased dietary restraint, and engagement in weight-loss strategies [8,9]. These perceptual distortions may be further reinforced by the dominant information sources used by young adults, in which exposure to inconsistent, emotionally charged, or misleading health-related content can influence beliefs about diet, body image, and weight-control strategies [36]. Such perceptual distortions may be amplified by sociocultural pressures and media exposure prevalent in university settings. Importantly, negative body image has been associated with lower self-esteem, heightened anxiety, and reduced cognitive performance, suggesting that body weight perception acts as a psychological mediator linking diet to emotional and cognitive health outcomes.

High caffeine intake and frequent consumption of energy drinks, often used to counteract sleep deprivation, have been linked to increased anxiety, irritability, and impaired sleep quality [37,38]. Although caffeine may temporarily enhance alertness, excessive intake disrupts sleep architecture and exacerbates stress-related symptoms, thereby indirectly influencing dietary choices and emotional stability [39,40]. Similarly, alcohol consumption, including binge drinking patterns, has been associated with depressive symptoms, impaired executive functioning, and long-term cardiovascular and neurological risks [41,42,43,44,45]. These behaviors may compound the negative effects of a poor diet on emotional and cognitive health.

Sleep quality emerged as a critical intermediary variable in the relationship between diet and mental health. Insufficient or poor-quality sleep has been shown to alter appetite-regulating hormones, increase hedonic eating, and impair cognitive functioning [14,15,16]. The findings of the present study support the existence of a self-perpetuating cycle in which academic stress and lifestyle behaviors disrupt sleep, which in turn negatively affects dietary habits, emotional regulation, and cognitive performance. Breaking this cycle may represent a key target for preventive interventions in students.

The role of physical activity and nutritional knowledge further contextualizes these findings. Previous studies have demonstrated that regular physical activity is associated with improved emotional well-being, enhanced cognitive function, and more favorable body image [18,19,20,46]. In contrast, sedentary behavior has been linked to increased cardiovascular risk and poorer quality of life among students [20,21,22,24]. Nutritional knowledge and diet self-management skills appear to buffer against unhealthy eating behaviors and distorted body weight perception, supporting the importance of educational strategies that integrate nutritional literacy with mental health promotion [27,47,48].

The increasing popularity of restrictive dietary strategies among students introduces additional complexity. Although approaches such as intermittent fasting or ketogenic diets may offer short-term metabolic benefits, their long-term psychological and cognitive effects remain controversial [36,37,38]. Extreme dietary restriction may exacerbate psychological distress, intensify stress-related responses, and negatively influence cognitive functioning, particularly in emotionally vulnerable individuals. Evidence suggests that anxiety and depressive symptoms may interact with maladaptive health behaviours and self-perception processes, thereby increasing susceptibility to unhealthy dietary patterns and impaired quality of life [49]. Moreover, dietary behaviours during critical developmental stages have been associated with broader health outcomes, including general somatic development and behavioural regulation, highlighting the multidimensional impact of nutrition on well-being [25]. These findings underscore the need for caution in promoting trend-driven diets without adequate consideration of their potential mental and cognitive health implications.

### Limitations and Future Directions

Several limitations should be acknowledged. The cross-sectional design precludes causal inference and relies on self-reported data. Additionally, psychological variables were assessed through self-perception rather than clinical diagnosis. Future research should adopt longitudinal designs to clarify causal pathways between diet, body weight perception, emotional health, and cognitive outcomes. Incorporating objective biomarkers of inflammation, metabolic status, and cognitive performance could further enhance understanding of the underlying mechanisms. Several additional limitations should be highlighted. First, participants were recruited via convenience sampling through online channels, which may have introduced selection bias and limited the sample’s representativeness to Romanian university students with internet access. Second, no multivariate modelling (e.g., logistic or linear regression adjusting for sex, age, and BMI) was performed; therefore, the reported associations are bivariate and may be partly explained by unmeasured confounders. Third, key psychological constructs (perceived stress, mood, body image) were assessed using single-item self-report measures rather than validated multi-item scales, which restricts the precision and construct validity of these variables. Fourth, sensitive behaviors such as restrictive dieting, self-induced vomiting, use of weight-control supplements, and prolonged fasting may be subject to social desirability bias, leading to under-reporting. Fifth, body mass index was derived from self-reported height and weight rather than objectively measured anthropometry, which is known to introduce systematic reporting bias. Finally, given the cross-sectional and observational nature of the data, all relationships should be interpreted as associations or correlations rather than causal effects; throughout the manuscript, terms such as “associated with” or “correlated with” are preferred over causal language.

Future studies may also explore targeted interventions combining nutritional education, stress management, and lifestyle modification to improve both mental well-being and cognitive functioning in student populations. Given the critical developmental nature of young adulthood, such interventions may yield long-term benefits extending beyond the academic context.

## 5. Conclusions

The present study highlights the multifaceted relationship between dietary habits, body weight perception, lifestyle behaviors, and psychological factors among university students. The findings reinforce the notion that eating behaviors during young adulthood are embedded within a broader biopsychosocial framework, in which emotional state, cognitive demands, self-perception, and daily routines interact dynamically to shape health-related outcomes.

Irregular eating patterns, unhealthy dietary choices, and engagement in restrictive dieting strategies were commonly observed and were correlated with increased psychological distress, altered body weight perception, and unfavorable lifestyle profiles. Discrepancies between calculated BMI (from self-reported height and weight) and subjective body weight perception emerged as a relevant factor influencing eating behaviors and emotional well-being, underscoring the importance of addressing perceptual and psychological dimensions alongside nutritional status. These findings support existing evidence that distorted body image may act as a mediator linking dietary behaviors to emotional and cognitive health.

Lifestyle factors such as sleep quality, physical activity, caffeine intake, and alcohol consumption further modulated the observed associations, often amplifying the negative effects of poor dietary habits. The interdependence of these behaviors suggests self-reinforcing cycles that may compromise both mental well-being and cognitive performance in academically demanding environments.

From a broader perspective, the results emphasize the need for integrative, multidisciplinary approaches to student health promotion. Interventions targeting dietary behaviors should be accompanied by strategies to improve nutritional literacy, emotional regulation, stress management, and healthy lifestyle practices. Universities represent a strategic setting for implementing such preventive programs, with the potential to yield long-term benefits extending beyond academic life.

In conclusion, understanding dietary habits in relation to body weight perception and psychological well-being is essential for promoting emotional balance and cognitive health during a critical developmental stage. Future research and public health initiatives should prioritize holistic models that recognize nutrition as a central component of mental and cognitive resilience among young adults.

## Figures and Tables

**Table 1:** Distribution of subjects according to gender and background.

Gender	Rural (%)	Urban (%)	Total (%)
Female	13.2	46.3	59.5
Male	7.4	33.1	40.5
Total	20.5	79.5	100

**Table 2:** Distribution of subjects according to age.

Age (Years)	Female (%)	Male (%)	Total (%)
Under 20	15.0	11.8	26.8
20–21	14.7	10.0	24.7
22–23	7.4	6.3	13.7
24–25	10.3	5.0	15.3
≥26	12.1	7.4	19.5
Total	59.5	40.5	100

**Table 3:** Distribution of caffeine consumption patterns among participants (multiple responses were permitted).

	% of Total
Natural coffee	59.74%
Instant coffee (Nescafé, soluble powders, capsules)	21.05%
Green/black tea	15.00%
Energy drinks (RedBull, Monster Energy, etc.)	11.32%
I don’t consume caffeine	25.00%

**Table 4:** Spearman correlation coefficients between selected lifestyle behaviours, dietary practices, and maladaptive weight-control behaviours among university students.

Variables	Correlation Coefficient (r)	*p*-Value
Variety of alcoholic beverages consumed vs. alcohol consumption frequency	0.734	<0.001
Sleep duration vs. perceived rest quality	0.364	<0.001
Calorie tracking vs. regular weight monitoring	0.120	0.019
Dieting practices vs. reported dieting success	0.816	<0.001
Intentional food restriction practices vs. supplement use	0.233	<0.001
Intentional food restriction practices vs. self-induced vomiting	0.274	<0.001
Supplement use vs. self-induced vomiting	0.387	<0.001

Note: This strong association should be interpreted cautiously because only participants who reported dieting could evaluate diet outcomes.

**Table 5:** The correlation between the media from which negative comments come.

Variables	Correlation Coefficient (r)	*p*-Value
External negative comments vs. family negative comments	0.523	<0.001
External negative comments vs. family support	−0.119	0.020
Family negative comments vs. family support	−0.313	<0.001

## Data Availability

The original contributions presented in this study are included in the article. Further inquiries can be directed to the corresponding authors.

## Supplementary Materials

The following supporting information can be downloaded at: https://www.mdpi.com/article/10.3390/nu18121837/s1.
