# Supplementary material for: Eating Habits, Body Weight Perception, and Psycho-Emotional Factors Among Romanian University Students: A Cross-Sectional Study
Source: Nutrients. 2026 Jun 6;18(12):1837. doi: 10.3390/nu18121837 (PMC13305504; doi:10.3390/nu18121837)
Supplement: Supplementary file 1 [file nutrients-18-01837-s001.zip › nutrients-4255860-supplementary.pdf]

Supplementary file

SECTION 1/7

Dietary Habits Among University Students

This questionnaire forms part of our research within a study focused on the topic of dietary habits. Through this study, we aim to obtain a comprehensive overview of dietary patterns among university students, supporting the understanding and management of potentially maladaptive eating behaviours.

Participation in this study is voluntary, and all responses are anonymous and confidential. The questionnaire takes a maximum of 10 minutes to complete and there are no right or wrong answers. The data collected will be processed for strictly academic purposes, and no identifying information is required.

Thank you for your time!

SECTION 2/7

PERSONAL DATA PROCESSING CONSENT AGREEMENT

In accordance with the requirements of Regulation (EU) 679/2016 on the protection of personal data of European Union citizens and on the free movement of such data, and repealing Directive 95/46/EC (the General Data Protection Regulation), as well as Law No. 506/2004 on the processing of personal data and the protection of privacy, the research team is obligated to manage, under secure conditions and solely for the specified purposes, the data you will provide: socio-demographic data and subjective responses.
As a participant in this study, I acknowledge that I have read and understood the personal data processing notice accompanying this consent form.

- YES
- NO (The questionnaire ends here)

SECTION 3/7

I. General and anthropometric data

1. Please select your gender from the box below:

- Female
- Male

2. Please enter your age: *(completed years)*

3. Area of residence:

- Urban
- Rural

4. Please indicate your field of activity:

- Health Sciences (medicine, pharmacy, dental medicine, etc.)
- Mathematics and Natural Sciences (IT, engineering, chemistry, physics, telecommunications, etc.)
- Social Sciences (law, sociology, political science, military sciences, economics, psychology, etc.)
- Humanities and Arts (language and literature, theology, history, arts, etc.)
- Sport Science and Physical Education (physical education, kinesitherapy, etc.)
- Other

5. What is your body weight? *(kg)*

6. What is your height? *(cm)*

7. Do you have any chronic conditions in your personal medical history? If yes, please specify the name of the condition:

II. Lifestyle characteristics

1. What type of diet do you follow?

- Predominantly carnivorous (meat as the dietary staple)
- Balanced omnivorous (both plant-based and animal-based foods)
- Ovo-lacto-vegetarian (plant-based foods, dairy products, and eggs)
- Vegetarian (plant-based foods only)
- Other

2. How many main meals do you consume per day?

- 1
- 2
- 3
- More than 3 main meals per day

3. How many snacks do you consume between main meals in a day?

- I do not consume snacks
- 1
- 2
- 3
- More than 3 snacks per day

4. At what time of day do you consume your first meal?

- In the morning, immediately or within 1 hour of waking up
- At midday
- Late in the day

5. Please indicate your weekly level of physical activity:

- Sedentary — no physical activity
- Light physical activity (1–2 times/week)
- Moderate physical activity (3–4 times/week)
- Intense physical activity (daily)

6. What type of physical activity do you prefer?

- Light exercise (walking)
- Moderate exercise (jogging, Pilates, etc.)
- Intense exercise (bodybuilding, swimming, tennis, etc.)

7. Please specify what type of caffeinated beverages you consume:

- Natural/ground coffee
- Instant coffee (Nescafé, soluble powders, capsules)
- Green/black tea
- Energy drinks
- I do not consume caffeine

8. How often do you consume caffeinated beverages?

- Once a day
- Twice a day
- Three times a day
- More than three times a day
- I do not consume caffeine

9. Do you regularly consume alcoholic beverages? *(over the course of a month)*

- Yes
- No

10. Please specify what type of alcoholic beverages you consume:

- Beer (including cider and carbonated alcoholic beverages)
- Wine
- Spirits (whisky, cognac, vodka, etc.)
- I do not consume alcohol

11. How often do you consume alcoholic beverages?

- Rarely (1–2 times/month)
- Occasionally (1–2 times/week)
- Frequently (2–3 times/week)

- Very frequently (3+ times/week)
- I do not consume alcohol

12. How many hours of sleep do you typically get?

- 7–8 hours
- 8–9 hours
- 9+ hours
- Fewer than 7 hours

13. How rested do you feel after a typical night's sleep?

- Well-rested
- Somewhat rested
- Not rested at all

14. How satisfied are you with your physical appearance?

- Very satisfied
- Satisfied
- Not satisfied at all
- Physical appearance is not important to me

15. Recently, have you experienced any of the following? *(multiple answers possible)*

- Stress
- Social anxiety
- Depression
- Sleep disturbances
- Suicidal ideation
- Concentration difficulties
- Excessive fatigue

SECTION 5/7

III. 3. Dietary habits and nutritional knowledge

1. Are you familiar with the difference between macronutrients and micronutrients?

- Yes, I have advanced knowledge on the subject
- Yes, I have intermediate knowledge on the subject
- No

2. When purchasing food, what do you pay attention to? *(multiple answers possible)*

- I read the label and nutritional declaration
- I only purchase brands I trust

- Texture, smell, or colour are important selection criteria for me
- Price is the most important factor
- These aspects do not matter to me

3. Do you regularly calculate your daily caloric intake requirements?

- Always
- Sometimes
- Rarely
- Never

4. How often do you weigh yourself?

- Every day
- Once a week
- Once a month or less
- I do not typically weigh myself

5. Have you ever followed a weight-loss diet? *(minimum 2 weeks)*

- Yes
- No

6. Please specify what type of diet you followed: *(multiple answers possible)*

- Intermittent fasting
- Ketogenic diet
- Paleo diet
- Detox/low-carb/very low-carb diets
- I have not followed any diet
- Other

7. What results did you achieve following the diet?

- I reached my goals and continued with the diet
- I reached my goals and returned to my usual dietary habits
- I was unable to adhere to the diet and discontinued it
- I have not followed any diet

8. Have you ever resorted to starvation or deliberate food restriction in order to lose weight or prevent weight gain?

- Yes
- No

9. Have you ever taken, without medical advice, pills, powders, or supplements intended for weight loss?

- Yes
- No

10. Have you ever self-induced vomiting or taken laxatives in order to prevent weight gain?

- Yes
- No

SECTION 6/7

IV. Psychological and perceptual factors

1. Have you ever received negative comments regarding your physical appearance that affected your self-esteem?

- Yes
- No

2. Have family members or close individuals ever directed humiliating words or expressions toward you?

- Yes
- No

3. Does the thought of gaining weight frighten you?

- Yes
- No

4. Do your family members or close individuals offer you support and encouragement?

- Yes
- No

5. Do you adapt easily to new situations or changes in routine?

- Yes
- No

6. Do you work well under pressure, or is stress a motivating factor for you?

- Yes
- No

7. Have you ever sought psychotherapy at least once?

- Yes
- No

SECTION 7/7
FINAL DE CHESTIONAR

Thank you for your time! Your contribution is a central component of the study we are conducting, and without it, the research would not have been possible.

For further discussion or any additional inquiries, please do not hesitate to contact us via email at:
